# Remodelling-informed evaluation of electrical discharge machining-textured and diamond-like carbon-coated titanium implants

**DOI:** 10.3389/fbioe.2026.1954906

**Published:** 2026-10-19

**Authors:** Chiqian Zhang, Janosch Schoon, Marion Wienecke, Jan Heeg, Tassilo-Maria Schimmelpfennig, Lisa Marie Rickerts, Georgi Wassilew, Frank Schulze

**Affiliations:** 1 Center for Orthopaedics, Trauma Surgery and Rehabilitation Medicine, University Medicine Greifswald, Greifswald, Germany; 2 The Fourth People’s Hospital of Guiyang, Guiyang, China; 3 Materion GmbH, Wismar, Germany; 4 Produktionstechnologie-Institut WAVE gGmbH, Wismar, Germany

**Keywords:** bone remodelling, diamond-like carbon, electrical discharge machining, osseointegration, osteoimmunology, titanium implants

## Abstract

The growing number of arthroplasties is linked to a rise in implant failures, with aseptic loosening being a significant factor in the need for revision surgeries. The long-term success of titanium implants regarding robust osseointegration depends not only on osteoblast-driven bone formation, but also on a balanced osteoimmune process, linking implant surface cues to coordinated bone remodelling. Using electrical discharge machining (EDM) and diamond-like carbon (DLC) as exemplars of topographical and physicochemical surface-engineering strategies, this review summarizes their generated surface cues and discusses how these cues may influence protein adsorption, osteoblast behaviour, macrophage activation, osteoclastogenesis, and bone-remodelling outcomes. Current evidence remains largely focused on osteogenic endpoints, whereas macrophage and osteoclast-specific data are still scarce. We therefore re-evaluate EDM and DLC surface modifications through an osteoimmunology framework and propose that future studies incorporate immune cell and remodelling-linked endpoints, as well as combined EDM/DLC interfaces, to support rational surface design for durable osseointegration.

## 1 Introduction

By the end of 2024, more than 3 million hip and knee arthroplasties had been recorded in the German Arthroplasty Registry. Aseptic loosening accounted for 21.0% of hip and 21.8% of knee revision surgeries, remaining the most prevalent reason (Grimberg et al., 2025). These data highlight the need to improve durable osseointegration at the implant–bone interface.

Although titanium and its alloys are widely used in orthopaedic implants, infection, peri-implant inflammation, wear-debris-induced osteolysis, and insufficient osseointegration can limit long-term survival (Jiang et al., 2023; Loi et al., 2016). These complications indicate that implant success is not determined only by bulk material properties, but also by cellular activity at the implant–tissue interface (Hanawa, 2019). Therefore, the implant surface should not be regarded merely as a passive structural boundary; rather, it acts as the first biological interface and governs bone remodelling and subsequent implant ingrowth. In consequence, contemporary strategies aim to improve implant ingrowth by utilization of surface modifications.

Conventional titanium surface modifications, including roughening, texturing, coatings, ion incorporation, and bioactive molecule immobilization, are usually designed as static surface cues that aim to enhance osteoblast adhesion, proliferation, and osteogenic differentiation (Huang et al., 2018; Bai et al., 2017; Ma et al., 2018; Long et al., 2020; Souza et al., 2018). However, osseointegration occurs within a dynamic biological environment. Implant ingrowth is therefore not simply the result of increased bone formation, but depends on the coordinated balance between bone formation and bone resorption (Zhao et al., 2022; Liu et al., 2025). This review focuses on how implant surface cues are recognized by cells and translated into osteoimmune responses that ultimately influence bone remodelling. In particular, electrical discharge machining (EDM) and diamond-like carbon (DLC) serve as exemplars to delineate the biological effects of topographical versus physicochemical surface cues. The interactive paradigm between implant surface engineering and the osteoimmune environment is conceptualized in (Figure 1A).

**FIGURE 1:**
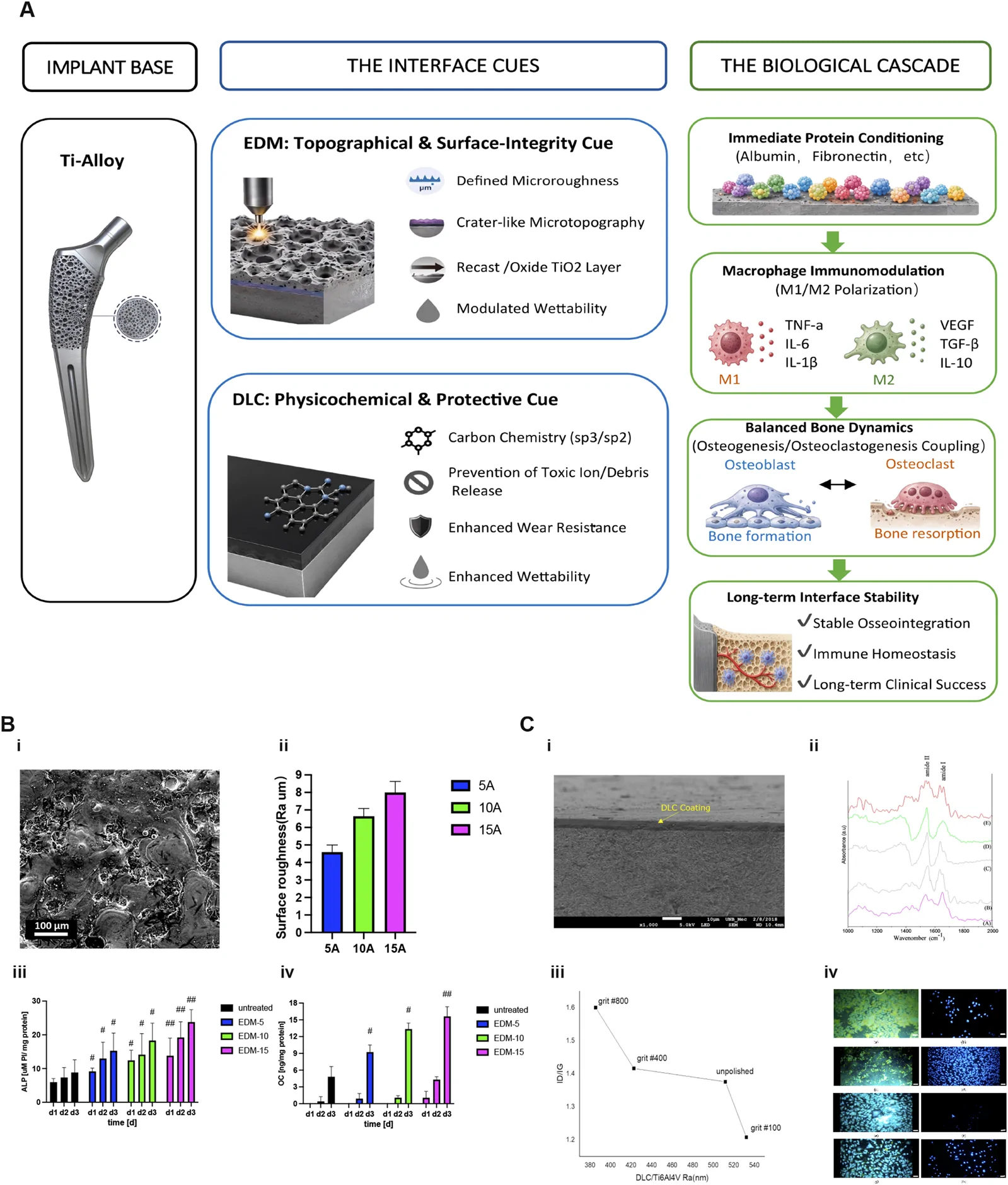
Proposed framework linking Ti-alloy surface modification to potential osteoimmune outcomes, supported by representative evidence for selected intermediate links. **(A)** Conceptual framework; **(B)** EDM-related evidence: (i) SEM image of EDM-modified Ti6Al4V surface, adapted from (Farooq et al., 2023); (ii) surface roughness of EDM-modified surfaces at different discharge currents; (iii, iv) ALP and osteocalcin levels under the corresponding EDM conditions. (# p < 0.05, ## p < 0.01 vs. untreated). Panels **(B)** (ii–iv) were digitized from (Yang et al., 2013); **(C)** DLC-related evidence: (i) scanning micrography of the cross-section of coated Ti alloy showing the DLC coating, reproduced from (Gobbi et al., 2024); (ii) Fourier-transform infrared spectroscopy (FTIR) spectra of free HSA and HSA adsorbed on DLC and Si-DLC surfaces (A–E: free HSA, DLC, SI, SII, and SIII, respectively), reproduced from (Ahmed et al., 2013); (iii) ID/IG ratios of DLC films on differently polished Ti6Al4V substrates; (iv) corresponding bright-field (left) and fluorescence (right) images of cultured cells: (a, b) unpolished, (c, d) #100-grit polished, (e, f) #400-grit polished, and (g, h) #800-grit polished. Panels **(C)** (iii, iv) were reproduced from (Wei et al., 2025).

## 2 From implant surface cues to osteoimmune remodelling

Osseointegration is a complex biological process that depends on coordinated cellular interactions at the implant–bone interface. After implantation, plasma proteins rapidly adsorb onto the implant and form a protein-conditioned layer. The protein composition of this adsorbed layer is influenced by material surface properties; in this way, material surface cues are first translated into a biological surface coating before being recognized by incoming cells. Macrophages shape the early inflammatory and regenerative microenvironment, while osteoblasts mediate matrix deposition and mineralisation, and osteoclasts participate in bone resorption. Thus, implant cues should be viewed as instructive signals driving early osteoimmune responses and subsequent remodelling.

### 2.1 Adhesion is an early event, not a remodelling endpoint

Early biological events at the implant interface include protein adsorption, cell adhesion, proliferation, and osteogenic marker expression. Cell adhesion represents merely an initial step, and these events collectively cannot reliably predict long-term implant stability or peri-implant remodelling. At this stage, cells interact mainly with this provisional protein layer rather than the bare titanium surface, using integrin-mediated recognition and downstream focal-adhesion signalling to initiate interfacial tissue integration (Gentleman and Gentleman, 2014; Nascimento et al., 2023; Barberi and Spriano, 2021). More importantly, this conditioned interface serves as a signalling hub for a diverse range of local cells, whose collective responses coordinate immune response, matrix deposition, resorption, and remodelling.

### 2.2 Macrophages as central regulators of osteogenesis

Following the initial neutrophil-dominated inflammatory response, monocyte-derived macrophages become the predominant immune regulators at the implant interface and shape the early cytokine environment. Tissue-resident bone macrophages (osteomacs) support bone formation by secreting osteogenic growth factors while also regulating the extracellular matrix environment (Miron and Bosshardt, 2016; Lin et al., 2020). Recruited macrophages and resident osteomacs together form a macrophage regulatory network in both temporal and spatial dimensions. This macrophage-dependent regulation of osteogenesis has been supported by both *in vitro* and *in vivo* studies. *In vitro* studies have reported that macrophage-conditioned media can alter the osteogenic differentiation of mesenchymal stromal cells (MSCs). Furthermore, macrophage-derived osteoimmune mediators, including interleukin-10 (IL-10) and oncostatin M (OSM), have been shown to enhance MSCs’ osteogenic responses under specific experimental conditions (Batoon et al., 2017; Schlundt et al., 2021). Consistent with these findings, *in vivo* studies have also shown that macrophage depletion leads to persistent neutrophil accumulation, reduced local T cell and MSC numbers, and impaired biomaterial integration (Morandini et al., 2024). In addition, depletion of osteomacs directly reduces collagen deposition and mineralisation, further demonstrating the functional support provided by osteomacs during osteogenesis and bone repair (Alexander et al., 2011).

In addition to cytokine-mediated regulation of osteogenesis, osteomacs maintain local microenvironmental homeostasis during bone remodelling through efferocytosis, the process that describes the phagocytotic removal of apoptotic cells. Following completion of their bone-forming function, most osteoblasts undergo apoptosis. If apoptotic cells are not efficiently cleared, the release of their intracellular contents can trigger local inflammatory responses and disrupt the orderly progression of bone remodelling. In turn, osteoblast apoptosis stimulates osteomac-mediated efferocytosis and promotes bone formation. Consistently, engulfment of apoptotic MSCs increased the secretion of bone regenerative factors (Batoon et al., 2024; Michalski et al., 2016).

### 2.3 Macrophages affect osteoclasts in bone remodelling

Beyond supporting osteogenesis and maintaining tissue homeostasis, macrophages not only serve as osteoclast precursors but also regulate osteoclastogenesis through their polarization state, receptor activator of nuclear factor kappa-B ligand (RANKL) signalling, the inflammatory environment, and phagocytic feedback mechanisms. Thus, they participate in bone remodelling at both the lineage and functional levels. Experimental studies have shown that pro-inflammatory M1 macrophages enhance osteoclast differentiation by increasing RANKL expression and pro-inflammatory cytokine production, thereby promoting bone resorption (Wei et al., 2005). In contrast, M2 macrophages inhibit osteoclastogenesis through the secretion of anti-inflammatory mediators (Luo et al., 2009).

This macrophage-regulated osteoclastogenic process subsequently feeds back to osteogenesis. Osteoclasts play a critical coupling role within this process. Beyond resorbing bone matrix, osteoclasts secrete factors that regulate osteoblast recruitment, differentiation, and bone formation, thereby linking controlled bone resorption with osteogenesis (Lotz et al., 2018). Evidence from high-turnover bone models further supports this coupling relationship. In osteoprotegerin (OPG) deficient and RANKL-transgenic mice, enhanced osteoclast activity is accompanied by increased bone formation. One proposed mechanism is that osteoclast-derived leukemia inhibitory factor (LIF) suppresses sclerostin expression in osteocytes, thereby reducing inhibition of the Wnt/β-catenin signalling pathway and promoting osteoblast-mediated bone formation (Udagawa et al., 2020).

Collectively, these findings indicate that osteoclast-mediated bone resorption is not merely a destructive process but mandatory for efficient bone remodelling. Therefore, implant evaluation should assess whether the surface supports a balanced osteoimmune remodelling network across different cells, rather than osteoblast activity alone.

### 2.4 Cell-specific interpretation of surface cues

The peri-implant multicellular framework raises a further question: how do different cell lineages interpret the same implant surface? Upon contact with biological fluids, the resulting protein layer composition is regulated by surface properties, including roughness, wettability, surface oxide chemistry, and coating composition. These parameters determine not only the quantity of adsorbed proteins, but also their spatial distribution, conformation, and bioavailability. Consequently, these surface properties define the cell-recognizable cues presented at the material interface and influence subsequent cellular responses.

Osteoblasts are the principal bone forming cells. They recognize adsorbed matrix proteins through α_1_β_1_, α_2_β_1_, and α_5_β_1_ integrins, activating focal adhesion kinase (FAK), and downstream extracellular signal-regulated kinase 1/2 (ERK1/2) and protein kinase B (AKT) networks (Krause et al., 2000; Raines et al., 2019; Zreiqat et al., 2002). The resultant F-actin remodelling amplifies cytoskeletal tension to drive the nuclear translocation of yes-associated protein (YAP), which ultimately dictates osteogenic marker expression, culminating in significantly enhanced ALP expression (Zhao et al., 2021).

The macrophage response to surface topographic cues is characterized by significant bidirectionality and context-dependence. Some literature indicates that rough surfaces can activate nuclear Factor kappa-light-chain-enhancer of activated B cells (NF-κB) mediated pro-inflammatory signalling through cell membrane deformation and integrin reorganization. These changes may polarize macrophages toward a pro-inflammatory M1 phenotype (Hu et al., 2024; Li J. et al., 2020; Pitchai et al., 2023). Conversely, relatively smooth surfaces tend to limit cell spreading and the extensive rearrangement of adhesion structures, which are generally associated with reduced cytoskeletal tension and mechanotransduction signalling. Such conditions have been reported to favour an anti-inflammatory or pro-reparative M2 phenotype (Özcolak et al., 2024). Notably, this polarization response is further refined by topographical features at the micro and nanoscale, with pitted and protrusive structures shown to preferentially promote M1-like polarization (Luu et al., 2015).

The effect of wettability on macrophage polarization exhibits a complex dependence, characterized by a dual driving mechanism in which both hydrophilic and superhydrophobic surfaces promote the M2 phenotype. Visalakshan et al. demonstrated that hydrophilic surfaces enhance the specific adsorption of albumin, creating an immunological microenvironment conducive to the release of anti-inflammatory factors (Visalakshan et al., 2019). However, the study also revealed that superhydrophobic surfaces can restrict cell spreading, inducing macrophages to form three-dimensional spheroids, thereby altering intracellular stress distribution and similarly guiding polarization toward the M2 phenotype (Del Carmen Moran et al., 2024).

This cell-specific interpretation of material surfaces suggests that evaluation should not rely solely on experiments performed with one cell type. A surface that supports osteoblast adhesion may still trigger an adverse macrophage response, whereas a surface with seemingly modest effects on osteoblasts directly may indirectly promote osteogenesis by inducing a pro-repair secretome. On this basis, it becomes necessary to define the engineering signatures of specific surface modification strategies.

## 3 Engineering signatures of EDM and DLC surfaces

Titanium alloys like Ti6Al4V and Ti6AlNb combine a high strength-to-weight ratio, favourable fatigue performance, corrosion resistance, and acceptable biocompatibility and are therefore the most widely used implant materials in musculoskeletal surgery (Chen et al., 2025; Falanga et al., 2019). However, titanium alloys also present engineering and biological limitations such as wear, corrosion, and ion release when exposed to bodily fluids. Generally, titanium alloys are considered to be bioinert materials. This means that they can support tissue contact mainly through surface-mediated interactions, but they have limited ability to form direct chemical bonding with surrounding bone without further surface activation (Barrak et al., 2022; Gaur et al., 2022; Mierzejewska et al., 2026; Toledano-Serrabona et al., 2022). These limitations have motivated the development of surface modification strategies that aim to improve stable long-term osseointegration of the implant.

Recent advances in implant engineering and materials processing have further emphasized the role of surface architecture and coating strategies in optimizing the biological performance of orthopaedic implants (El-Bassyouni et al., 2025; Wu et al., 2026). Conventional strategies to optimize the titanium interface encompass a broad spectrum of topographical texturing, barrier coatings, and biochemical functionalization. Within this landscape, EDM and DLC coatings represent examples for topographical and physicochemical engineering, respectively. While EDM modulates microstructural roughness, DLC provides chemical inertness and tribological protection (Singh et al., 2025; Yick et al., 2023). Both strategies have been investigated for their effects on implant biocompatibility and osteogenic response. Accordingly, this section defines how EDM and DLC generate distinct surface cues and synthesizes the experimental evidence for their biological performance in orthopaedic-relevant models.

### 3.1 EDM: topographical and surface-integrity modification

EDM is a non-traditional electro-thermal paradigm that utilizes controlled sparks within a dielectric medium to induce localized thermal erosion (Joshi and Joshi, 2019). For titanium processing, this fundamental mechanism has diversified into advanced platforms for surface feature control (micro-EDM), dielectric chemistry (powder-mixed and near-dry EDM), and field-assisted coupling (ultrasonic and magnetic hybrids) (Abu Qudeiri et al., 2019; Pramanik et al., 2020). Despite process differences, they are all derived from electrical-discharge-induced thermal erosion of conductive materials. Representative EDM-modified surface morphology is shown in (Figure 1Bi) (Farooq et al., 2023).

During each discharge, localized melting, evaporation, and rapid re-solidification of the machined material occur, thereby generating a distinct modified layer *in situ*. Consequently, EDM does not merely shape the geometry of conductive metals; it serves as an effective method for constructing a multifunctional, engineered surface topography. For titanium and its alloys, this process can generate a crater-like microporous topography, promote the incorporation of oxide or carbide phases, and significantly alter surface wettability and microhardness by precisely controlling discharge parameters and the dielectric fluid. The resulting interfacial layer is of paramount importance for load-bearing biomedical implants. It serves as the primary contact zone resisting complex electrochemical degradation and cyclic mechanical loading, while simultaneously mediating early interactions with proteins, physiological fluids, and cells (Nafi and Jahan, 2022). These key material features are summarized in (Table 1A).

**TABLE 1:** Engineering signatures and potential biological relevance of EDM- and DLC-modified titanium surfaces.

Feature Dimension	Reported Parameters	Typical EDM effects and sources	Possible biological correlations	References
A. EDM-modified titanium surfaces				
Micro-morphology	Ra: 10.8–17.6 μm; Rz: 85.3–167.4 μm (high-power EDM of Ti-6Al-4V ELI); crater diameter and depth; other Rt, Rv, Rq, Rsm	Discharge energy, current, and pulse-on time (T-on) affect crater depth and recast material, and significantly affect Ra and Rz	Roughness and regular crater arrays determine protein adsorption-site density and cell-spreading contact area. Controlled microroughness is commonly used to improve bone anchoring and osseointegration	(Karmiris-Obratanski et al., 2021; Hess et al., 2024; Rehman et al., 2022; Kiyak, 2022; Le Guehennec et al., 2007)
Surface Chemistry	Oxide fraction (e.g., TiO_2_), Titanium Carbide, O/Ti ratio; alloying or powder-derived elements such as SiC, and Al, Cu, Ag	Thermal oxidation and plasma decomposition generate oxide-rich layers. Dielectric fluid, electrode, and powder decomposition can form oxides and carbides. Bioactive phases such as HAP and TiO_2_ can be produced	Surface chemistry may affect competitive protein adsorption, corrosion or degradation rates, and osteoconductivity or bioactivity. Ca-P and Si, elements enrichment may be associated with improved osteoblast adhesion and proliferation	(Barberi and Spriano, 2021; Li et al., 2019; Srivastava et al., 2023; Öpöz et al., 2018; Alam et al., 2021; Calderon et al., 2022; Nauryz et al., 2023; Huang et al., 2026; Bui et al., 2020)
Wettability	Static contact angle (θ): 30.2 ± 1° (untreated Ti-6Al-4V), 0–17.8° (after EDM treatment); surface free energy	Several studies report EDM and powder-mixed EDM surfaces processed under suitable parameters show lower contact angles and improved hydrophilicity	Hydrophilic surfaces favour early protein adsorption and cell adhesion	(Jabbaripour et al., 2012; Rupp et al., 2014; dos Santos et al., 2025)
Surface Integrity	Recast layer thickness; residual stress; micro-crack and pore density	Increased discharge energy leads to thicker white layers and more cracks or pores	Overly thick white layers with micro-cracks are linked to lower fatigue strength, local corrosion, and long-term stability risks	(Zhu et al., 2020; Mahdieh and Mahdavinejad, 2018)

Feature Dimension	Reported Parameters	DLC-induced engineering signature	Possible biological correlations	References
B. DLC-midified titanium surfaces				
Carbon bonding structure and surface chemistry	sp3/sp2 ratio: 1.5–2.4, ID/IG: 1.6–2.5; G-peak position; hydrogen content; C content; XPS C–C/C–H/C–O composition; F, Si, Ti, Ag, Cu, Cr or other dopants	DLC is an amorphous carbon coating whose properties—hardness, inertness, wear resistance, conductivity, and friction—depend on its sp^3^/sp^2^ ratio, hydrogenation, and doping	Different sp^3^/sp^2^ structures affect protein adsorption, cell spreading, and debris behaviour, while elemental doping can enhance antibacterial properties	(Dwivedi et al., 2011; Zhang et al., 2020; Zemek et al., 2021; Shang et al., 2023; Lanzerstorfer et al., 2024; Sun et al., 2023)
Surface energy, wettability	Water contact angle: 68.9 ± 0.4° (untreated Ti-6Al-4V), 77.4 ± 0.2° (after DLC treatment); surface free energy; polar/dispersive components; zeta potential	DLC can alter titanium surface energy and wettability. The effect is not fixed: different DLC chemistries and dopants can produce different contact angles and surface free-energy profiles	Wettability affects the early adsorbed protein layer	(Huang et al., 2022; Fujii et al., 2024; Tong et al., 2025; Jongwannasiri et al., 2023)
Mechanical, tribological and corrosion-protective function	Hardness; elastic modulus; H/E ratio: 0.038–0.14; wear rate: 4.30 × 10^−4^ mm^3^·N^−1^·m^−1^ (untreated Ti-6Al-4V), 2.97 × 10^−8^ mm^3^·N^−1^·m^−1^ (after DLC treatment); wear-track morphology	DLC is mainly valued as a hard, low-friction, wear-resistant and corrosion-resistant coating	By reducing wear debris, corrosion products and metal ion release, DLC may indirectly mediated chronic inflammation, foreign-body response	(Malisz et al., 2023; Zhang et al., 2024; Madej et al., 2025)
Coating integrity and long-term stability	Coating thickness; adhesion strength; residual stress; delamination	DLC performance depends on stable coating adhesion and high residual stress; weak adhesion, cracking or delamination may compromise the tribological and barrier functions of DLC.	Coating integrity is a key limitation for load-bearing orthopaedic use	(Sun et al., 2023)

Abbreviations: ELI, extra low interstitials; G-peak, graphitic carbon Raman peak; H/E ratio, hardness-to-elastic modulus ratio; HAP, hydroxyapatite; ID/IG ratio, Raman D-band to G-band intensity ratio; Ra, arithmetical mean roughness; Rq, root mean square roughness; Rsm, mean spacing of profile irregularities; Rt, total profile height; Rv, maximum valley depth; Rz, maximum height roughness; sp^3^/sp^2^ ratio, ratio of tetrahedral to graphitic carbon bonding; XPS, X-ray photoelectron spectroscopy.

EDM-induced surface modification requires careful balancing of secondary effects. Higher pulse current and discharge duration promote recast-layer formation and microhardness but can also increase microcracks, blowholes and pitting (Jabbaripour et al., 2012; Nair et al., 2019). Although EDM tailors beneficial surface topography and chemistry, resulting defects may compromise surface integrity and fatigue performance in load-bearing implants. Precise control of processing parameters is therefore required, as these determine final surface characteristics and subsequent biological responses (Kou et al., 2025; Chen et al., 2026).

### 3.2 Biological evaluation of EDM-modified implant surfaces for orthopaedic applications

Based on these physical and chemical characterisations, the biological studies initially focused on cytocompatibility and osteogenic potential. Across studies, EDM-modified Ti6Al4V was associated with improved osteoblast proliferation, enhanced fatigue resistance, and no overt cytotoxicity (Havlikova et al., 2014; Kumar et al., 2022).

In addition to the confirmation of basic cytocompatibility, a few studies explored osteogenic responses to EDM-treated surfaces at an early stage. Wire-cut EDM-induced crater-like microtopography and submicron structures promote early osteoblast adhesion but inhibit late-stage proliferation, while significantly upregulating osteogenic genes (Otsuka et al., 2012). Similarly, higher EDM discharge parameters enhanced ALP activity, osteogenic cell growth, and nodule formation (Lee et al., 2013; Harcuba et al., 2012). Rougher EDM surfaces produced at higher currents also increased ALP and osteocalcin levels (Figure 1Bii–iv). (Yang et al., 2013) Despite variations in EDM processing methods across studies, most studies consistently have reported enhanced osteogenic activity on EDM-treated titanium surfaces.

Going a step further, Kurosaka et al. provided mechanistic clues in primary osteoblasts: the amorphous TiO_2_ layer generated by EDM may upregulate Hydrogen peroxide-inducible clone 5 (Hic-5) and osteogenic gene expression, thereby participating in collagen crosslinking and osteogenic matrix maturation (Kurosaka et al., 2022). The evidence suggests that EDM-modified surface features may support osseointegration, but whether and how specific features influence osteoimmune crosstalk and coordinated bone remodelling remains largely unknown, and the underlying mechanistic links remain speculative. Further studies focusing on key aspects, such as RANKL/OPG signalling and secretome profiling, would therefore be valuable before broad orthopaedic application.

### 3.3 DLC: physicochemical and tribological interface modification

DLC coatings are amorphous carbon films containing mixed sp^2^- and sp^3^-hybridized bonds, with properties between graphite and diamond. In contrast to topographical surface modification methods such as EDM, the effect of DLC is not primarily mediated by generating micro-scale surface craters or roughness. Rather, it functions as a thin physicochemical interface that changes the outermost chemistry, surface energy, tribological behaviour, and ion-barrier properties of metallic implants at their surface (Malisz et al., 2023; Mou et al., 2025) (Figure 1Ci). shows a representative cross-section of DLC-coated titanium alloy (Gobbi et al., 2024).

The biomedical relevance of carbon-based implant surfaces was recognized early. In 1977, Bokros demonstrated the successful use of carbon materials in prosthetic devices, supporting their biocompatibility (Bokros, 1977). In 1991, Thomson et al. found no adverse cellular effects of DLC-coated surfaces in mouse peritoneal macrophages and fibroblasts, suggesting that DLC deserved further investigation as a biomedical coating, particularly for orthopaedic implants (Thomson et al., 1991).

DLC is attractive for load-bearing implant applications, owing to its ability to optimize tribological performance and biocorrosion resistance (Hassan et al., 2023). By tailoring surface chemistry, bonding structure, and topography, DLC coatings modulate the conformation and organization of adsorbed proteins, which in turn facilitates the formation of a protective tribofilm and significantly extends long-term material durability (Shaikh et al., 2024). In one study, increasing Si incorporation into DLC was associated with increased human serum albumin (HSA) adsorption (Figure 1Cii). (Ahmed et al., 2013)Consequently, rather than a mere surface treatment, DLC is better framed as a functional physicochemical interface. These key material features are summarized in (Table 1B).

However, DLC coatings are also limited by their material properties. A higher sp^3^ fraction generally improves hardness and wear resistance, but it may also increase intrinsic residual stress, thereby compromising coating adhesion (Liu et al., 2018; Li C. et al., 2020). Accordingly, DLC design requires balancing mechanical performance, surface chemistry, biological compatibility, and long-term interfacial stability.

### 3.4 Biological evaluation of DLC modifications for orthopaedic implant surfaces

DLC coatings in orthopaedic applications have been limited by poor adhesion to metallic substrates and delamination risk in physiological fluids under mechanical loading (Skjoldebrand et al., 2022). To improve adhesion while tuning biological responses, subsequent DLC studies have used interlayers, elemental doping and sp^2^/sp^3^ control. Travnickova et al. showed that metal-doped DLC (Cr, Ti) supported stem cell behaviours relevant to bone repair beyond short-term cytocompatibility (Travnickova et al., 2024; Travnickova et al., 2020). Copper or phosphorus doping enhances DLC films, integrating electrochemical protection and antimicrobial barrier properties with improved osteoblastic cytocompatibility (Milan et al., 2020; Jastrzębski et al., 2024).

The biological response to DLC coatings is also influenced by their internal bonding structure. A lower ID/IG ratio, indicative of a more sp^3^-rich DLC structure, coincided with improved cell adhesion (Figure 1Ciii–iv) (We et al., 2025). Similarly,DLC films with reduced sp^2^/sp^3^ ratio promoted a more anti-inflammatory cytokine profile. This suggests that a denser, highly cross-linked diamond-like carbon network effectively modulates the local immune environment to suppress pro-inflammatory signalling (Liao et al., 2016). In addition to the sp^2^/sp^3^ bonding ratio, hydrogen incorporation represents another structural variable that can modify the biological performance of DLC. Mine et al. reported that specific hydrogenated DLC surfaces increased type I collagen mRNA expression in osteoblasts while suppressing Tartrate-Resistant Acid Phosphatase (TRAP) mRNA expression in osteoclast precursors (Mine et al., 2014). These *in vitro* findings suggest that DLC structure may modulate both inflammatory responses and bone remodelling.

Current research is moving toward the exploration of mechanistic pathways. In an ovine implantation model, Si-DLC implantation involved macrophage responses without triggering a clear systemic pro-inflammatory disturbance (Szymczak et al., 2024). In a tendon adhesion model, PLA/DLC membranes reduced ROS production, inhibited NF-κB activation and M1 polarization, and decreased pro-inflammatory cytokine expression (Xiao et al., 2024). Although most evidence is based on non-bone models, these studies indicate that DLC-based interfaces may modulate macrophage-associated inflammatory signalling. Overall, current evidence suggests that DLC coatings may create a biologically favourable bone–implant interface, but mechanistic evidence linking surface cues to immune-mediated remodelling remains limited.

## 4 Discussion and perspectives

The available evidence supports EDM and DLC as biologically active interfaces, but it does not yet establish how defined surface parameters are converted into osteoimmune and remodelling outcomes. Current evidence indicates that physical cues such as surface roughness can enhance osteoblast adhesion and differentiation. However, these responses are often accompanied by concurrent changes in surface properties, making it difficult to determine the relative contribution of individual surface parameters. More importantly, current studies provide limited mechanistic insight into how surface-derived signals are translated into cell-specific biological responses that ultimately shape the peri-implant remodelling microenvironment. For advanced surface modifications such as EDM and DLC, biological evaluation remains largely focused on conventional osteogenic endpoints, whereas direct evidence regarding macrophage and other immune-cell responses is still scarce. If a surface that has been found to enhance osteoblast activity, yet inadvertently activates pro-inflammatory macrophages or promotes osteoclast-mediated resorption, it could still lead to chronic inflammation or fibrous encapsulation.

In this context, the potential combination of EDM and DLC should be regarded as a promising interface-design concept. EDM and DLC represent complementary categories of surface cues, but complementarity at the engineering level does not automatically imply biological synergy. After confirming the physicochemical features of each treatment, the combined EDM/DLC surface should be re-characterized to verify that these individual surface functionalities are retained after hybridization. In particular, the interfacial adhesion and mechanical stability of the DLC coating on EDM-modified substrates should be evaluated, together with the potential effects of residual thermal stresses on interface stability. Protein conditioning and relevant biological responses should also be assessed on the hybrid surface, including osteoblast bone-forming activity, macrophage phenotype, cytokine secretion, and osteoclast bone-resorbing activity. The main value of such studies would not be simply to identify a better surface, but to clarify how topographical and physicochemical cues interact in regulating protein conditioning, immune-cell behaviour, osteoclast activity, and bone-remodelling outcomes and thus ultimately govern implant ingrowth.

In conclusion, to advance from surface bioactivity to remodelling-informed evaluation, future research must move beyond isolated osteoblast assays to integrate immune cells, osteoclasts, and bone resorption-formation coupling. Mechanistic studies directly assessing immune-mediated responses to EDM and DLC surfaces are needed, while broader implant design may benefit from the coordinated optimization of surface modification, material composition, and topological architecture to achieve long-term implant performance (Yuan et al., 2025).
